# Street rabies virus causes dendritic injury and F-actin depolymerization in the hippocampus

**DOI:** 10.1099/vir.0.047480-0

**Published:** 2013-02

**Authors:** Yan Song, Jinli Hou, Bin Qiao, Yanchao Li, Ye Xu, Ming Duan, Zhenhong Guan, Maolin Zhang, Liankun Sun

**Affiliations:** 1 Department of Pathophysiology, Norman Bethune College of Medicine, Jilin University, Xinming Road, Changchun 130021, PR China; 2 Key Laboratory of Zoonoses, Ministry of Education, Institute of Zoonoses, Jilin University, 5333 Xian Road, Changchun 130062, PR China; 3 Nursing College, Beihua University, 3999 Huashan Road, Jilin 132013, PR China; 4 Department of Histology and Embryology, Norman Bethune College of Medicine, Jilin University, Xinming Road, Changchun, 130021, PR China; 5 Medical Research Laboratory, Jilin Medical College, Jilin Road, Jilin 132013, PR China

## Abstract

Rabies is an acute viral infection of the central nervous system and is typically fatal in humans and animals; however, its pathogenesis remains poorly understood. In this study, the morphological changes of dendrites and dendritic spines in the CA1 region of the hippocampus were investigated in mice that were infected intracerebrally with an MRV strain of the street rabies virus. Haematoxylin and eosin and fluorescence staining analysis of brain sections from the infected mice showed very few morphological changes in the neuronal bodies and neuronal processes. However, we found a significant decrease in the number of dendritic spines. Primary neuronal cultures derived from the hippocampus of mice (embryonic day 16.5) that were infected with the virus also showed an obvious decrease in the number of dendritic spines. Furthermore, the decrease in the number of dendritic spines was related to the depolymerization of actin filaments (F-actin). We propose that the observed structural changes can partially explain the severe clinical disease that was found in experimental models of street rabies virus infections.

## Introduction

Rabies virus (RV) is a neurotropic virus that primarily targets the central nervous system (CNS). The deadly RV produces a variety of nervous system symptoms; however, patients eventually die of circulatory insufficiency (Hemachudha *et al.*, 2002). In contrast to the dramatic and severe clinical manifestations related to neuronal dysfunctions, only mild lesions in the CNS are observed during post-mortem examinations. Previous studies had demonstrated that the fetal rabies caused neuronal dysfunction, including ion channel dysfunction and neurotransmitter abnormalities rather than neuronal damage (Bouzamondo *et al.*, 1993; Iwata *et al.*, 1999; Tsiang, 1993), and that the infection with silver haired bat rabies virus resulted in the downregulation of several proteins that were relevant to synaptic physiology. Furthermore, the downregulation of these proteins can block synaptic vesicles from docking and fusing to the plasma membrane; therefore, the release and uptake of neurotransmitters is reduced (Dhingra *et al.*, 2007).

Dendrites (the branched projections of a neuron) and dendritic spines constitute major post-synaptic sites for excitatory synaptic transmission (Harris, 1999; Hering & Sheng, 2001; Sheng, 2001). Dendritic spines are highly motile and can undergo remodelling; in addition, their structural plasticity is tightly coordinated with synaptic function (Kasai *et al*., 2010). Spine loss or alteration has been described in patients with neurodegenerative diseases, such as Alzheimer’s disease (Fiala *et al.*, 2002; Law *et al.*, 2004). However, few studies have addressed if the neuronal dysfunction observed in the street rabies virus infection was associated with dendritic spine plasticity.

In this study, we used *in vivo* and *in vitro* methods to investigate the morphological changes of dendrites and dendritic spines in the hippocampus of mice that were infected by an MRV strain of the street rabies virus (GenBank accession no. DQ875050.1). The results showed that the RV infection decreased the number of dendritic spines. Further analyses confirmed that the dendritic changes were related to the depolymerization of filamentous actin (F-actin), a cytoskeleton protein that helps to regulate the morphogenesis and dynamics of dendritic spines (Fischer *et al.*, 2000; Matus *et al.*, 2000).

## Results

### Distribution of MRV antigen in the CNS

**Fig. 1.**
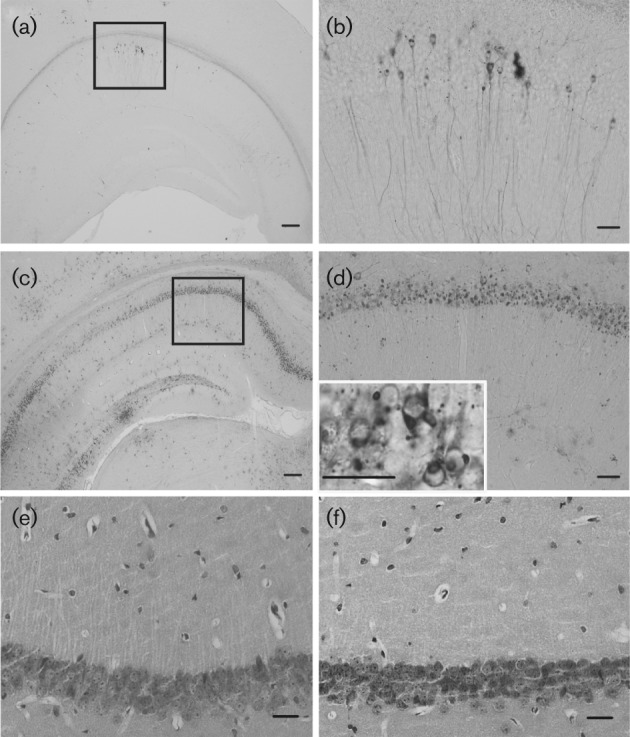
Immunohistochemical analysis of antigen distribution in the CA1 region of the hippocampus of mice infected with an MRV strain of the street rabies virus. The approximate Bregma level for the sections is −2.18 mm (a–d). (a, b) The RV antigen was detected in the cell bodies and processes of pyramidal neurons in the CA1 region of the hippocampus at day 4 p.i. (c, d) At day 7 p.i., the RV antigen was detected primarily in the neuronal somata, but decreased dramatically in the processes. (e, f) H&E staining showing that no obvious morphological changes are seen in the cell bodies or dendritic processes in the CA1 region of the hippocampus [control (e), MRV infection (f)]. Bar, 200 µm (a, c); 50 µm (b, d) and 10 µm (d, inset); 20 µm (e, f).

The MRV antigen has been detected in numerous brain regions, such as the cortex, the thalamus and the cerebellum (data not shown). In the hippocampus, MRV expression began to appear in the CA1 region at day 4 post-infection (p.i.), and viral antigen was seen in the perikarya and the intact processes of hippocampal pyramidal neurons (Fig. 1a, b). By contrast, almost no positive staining could be found in the neuronal processes at day 7 p.i., and the antigen was located mainly in the perikarya (Fig. 1c, d). At day 7 p.i., the mice became moribund, and a greater number of variably sized RV-positive inclusion bodies were found to appear in the infected areas (the inset in Fig. 1d).

### Morphological changes to cell bodies and dendrites in the CA1 region of the hippocampus

**Fig. 2.**
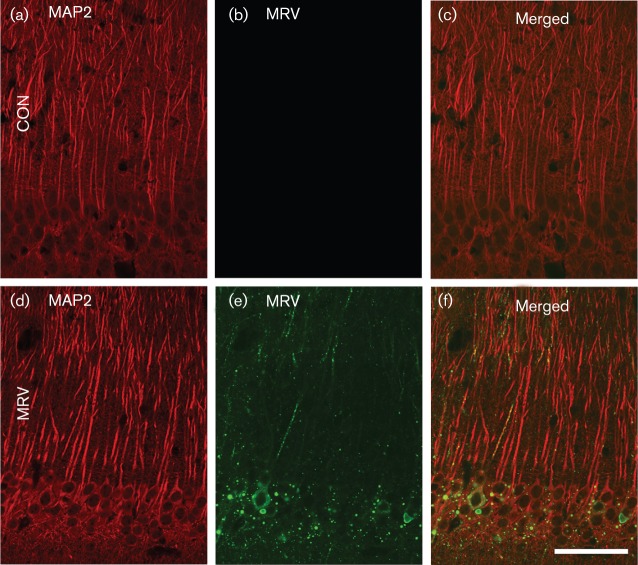
Immunohistochemical analysis of MAP2 (a, d) and MRV (b, e) in the CA1 region of the MRV-infected hippocampus at day 7 p.i. Although the RV antigen is detected primarily in the neuronal somata, fluorescence staining for MAP2 shows that both the cell bodies and the dendrites remained intact in the infected area (d). Con, Control. Bar, 50 µm.

The changes that occurred to cell bodies and dendrites in the hippocampus after MRV infection were studied by both conventional histological staining and immunolabelling using microtubule-associated protein 2 (MAP2), a neuronal cytoskeleton protein. As shown in Fig. 1(e, f), haematoxylin and eosin (H&E) staining of paraffin-embedded tissue sections did not demonstrate well-defined cytopathological changes in MRV-infected neurons. Similarly, fluorescence staining for MAP2 suggested that both the cell bodies and the dendrites remained intact in the infected area, even at the late infection stage (Fig. 2).

#### MRV infection decreased the number of dendritic spines *in vivo*.

**Fig. 3.**
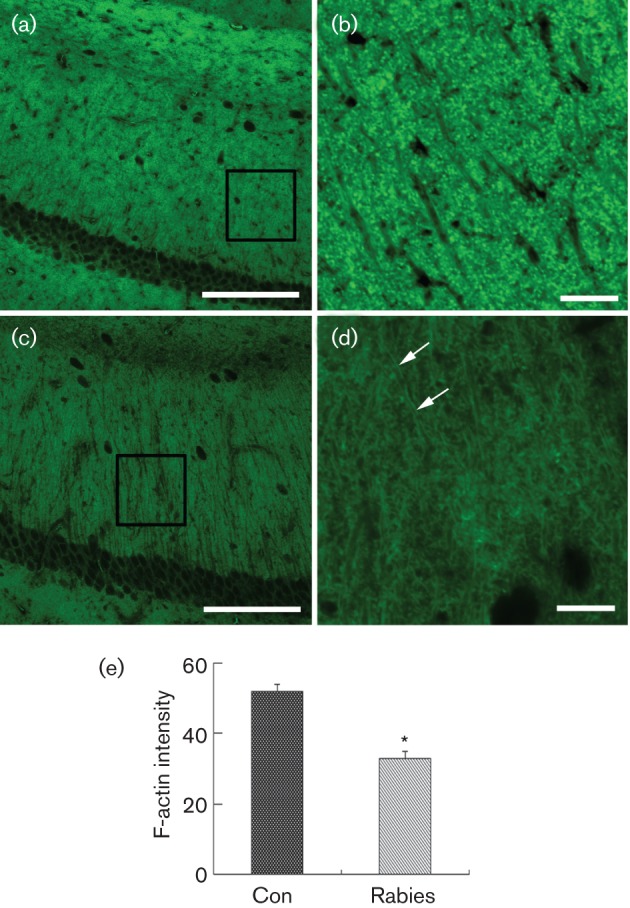
The decrease of F-actin in the CA1 region of the MRV-infected hippocampus at day 7 p.i. (a, b) In PBS-injected mice, fluorescence staining of F-actin with Alexa Fluor 488 phalloidin revealed numerous positive punctates in the striatum radiatum of the CA1 region, which is typical of the localization of F-actin in dendritic spines. The inset in (a) is enlarged in (b), showing the details of the dendritic spines. (c, d) The staining intensity and the number of positive punctuates dramatically decreased in MRV-infected mice. The inset in (c) is enlarged in (d). Additionally, many rope-like structures (arrows) appear along the dendrites in the brains of the injected mice (d), indicative of a reorganization of F-actin occurring post-synaptically. (e) F-actin intensity (arbitrary units) is significantly lower in the rabid mice (b) than in the control (Con) group (a) (*P<0.01, *n* = 10, Student’s *t*-test). Bar, 50 µm (a, c); 10 µm, (b, d).

Alexa Fluor 488 phalloidin conjugate was used to show the dendritic spines in the CA1 region of the hippocampus. The punctate labelling of phalloidin, which is typical for the location of F-actin in dendritic spines (Capani *et al.*, 2001), was observed in the PBS-injected mice (Fig. 3a, b). By contrast, a significant decrease in fluorescence intensity and punctate labelling was observed in the MRV-infected mice (Fig. 3c–e). In addition, many rope-like structures were observed to appear along the dendrites in the brains of the injected mice (arrows in Fig. 3d), indicative of a reorganization of F-actin occurring post-synaptically.

#### MRV infection decreased the number of dendritic spines *in vitro*.

To confirm our *in vivo* results, cultures of primary hippocampal neurons were prepared. At 28 days after plating, nearly 95 % of the cells isolated from the hippocampus expressed the neuronal marker MAP2 as assessed by using immunofluorescence staining indicating that they were neurons (data not shown). These neurons also exhibited a well-developed network and a high level of growth, indicative of a healthy and viable culture.

**Fig. 4.**
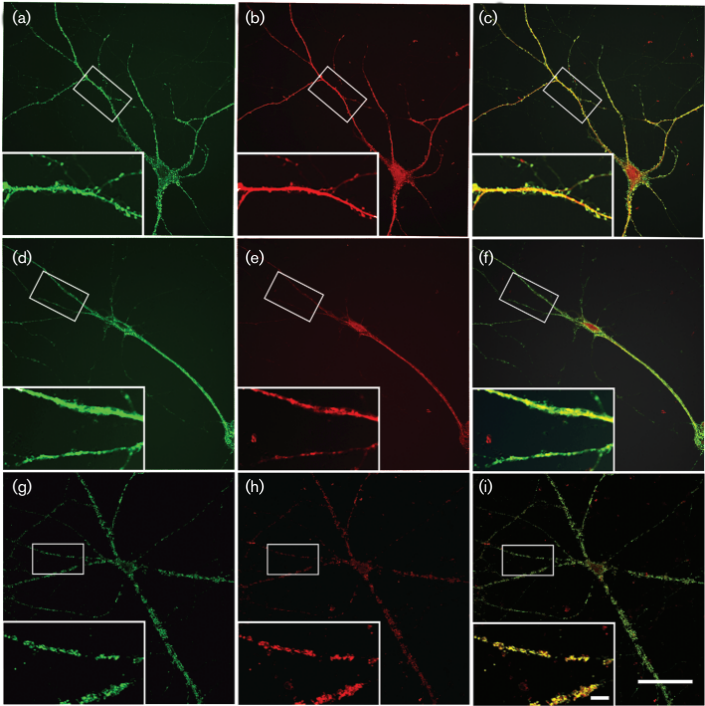
MRV infection decreases the number of dendritic spines in primary hippocampal neurons. At 28 days after plating, the primary hippocampal neurons were infected with MRV at a concentration of 10 TCID_50_. At day 4 (d–f) and day 6 (g–i) p.i., both the MPV-infected and mock-infected cells (a–c) were fixed, and stained for F-actin and MAP2. The inset in each panel is enlarged showing the details of the dendritic spines. (d–i) Infected neurons showed a decrease in the number and size of spines. When compared with those at day 4 p.i. (d–f), there was no continuous staining in the cell body or dendrites for either F-actin or MAP2 in infected neurons at day 6 p.i. Results shown are representative of experiments that were repeated four times. Bar, 50 µm (a–i); 15 µm (insets).

Alexa Fluor 488 phalloidin staining revealed that most pyramidal hippocampal neurons showed mushroom-like structures 28 days after plating, suggesting the presence of well-defined dendritic spines. Infection with MRV resulted in a dramatic decrease in the number and size of spines, as revealed by phalloidin staining. When compared with those at day 4 p.i. (Fig. 4d–f), the infected neurons appeared seriously damaged at day 6 p.i. and continuous staining was not found in their cell bodies or dendrites for either F-actin or MAP2 (Fig. 4g–i). Due to the presence of positive punctuates in the dendritic cytoplasm, spines could not be unequivocally distinguished at this infection stage, therefore only the changes of spines occurring at day 4 p.i. were examined and quantified. A significant decrease in the number of spines was found in the infected cells (4±2.0 µm^−1^, *n* = 12) compared with those in the control (12±3.0 µm^−1^, *n* = 10; *P*<0.01, Student’s *t*-test) (Fig. 4).

### Depolymerization of F-actin by MRV

**Fig. 5.**
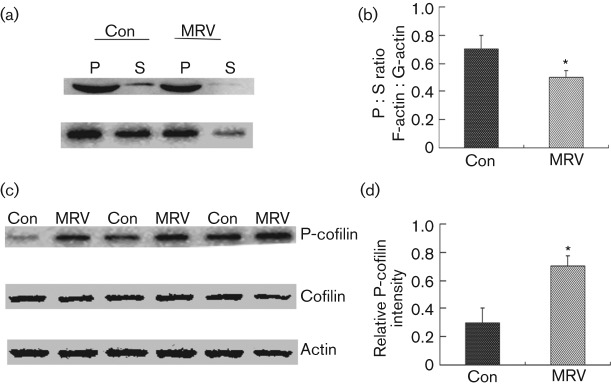
RV infection causes depolymerization of F-actin. (a) Western blot analysis of soluble actin (G-actin) in the supernatant fraction (S) and insoluble actin (F-actin) in the pellet (P) fraction of the hippocampus. The two centrifugation fractions from each sample were proportionally loaded, and the ratio of F-actin to G-actin was calculated (b). RV infection caused significant depolymerization of F-actin (*P<0.01, *n* = 6, Student’s *t*-test). (c) Western blot analysis of p-cofilin, total cofilin, and total actin in the hippocampi of the PBS-injected (control, Con) and MRV-infected (MRV) mice (three different animals each). (d) The ratio of p-cofilin to total cofilin (*P<0.01, *n* = 10, Student’s *t*-test).

The intensity of F-actin and G-actin in mice infected by MRV was analysed by Western blotting and the ratio of F-actin to G-actin was calculated. The intensity (*P*<0.01, *n* = 6–8 sections per mouse, *n* = 10 mice) and ratio (*P*<0.01, *n* = 6 mice per group, Student’s *t*-test) were significantly decreased in the CA1 region of the infected mice when compared with that in the PBS controls (Fig. 5a, b). It has been suggested that the polymerization or depolymerization of F-actin is regulated by the phosphorylation state of cofilin (Allison *et al.*, 1998; Capani *et al.*, 2001; Kuriu *et al.*, 2006; Renner *et al.*, 2009). Thus, we examined if the depolymerization of F-actin (caused by RV infection) was related to a decrease in the phosphorylated form of cofilin (phospho-cofilin, p-cofilin). Western blot analysis revealed that RV infection has no effect on the total levels of actin and cofilin; however, it can increase the levels of p-cofilin in the hippocampi of furious rabid mice (Fig. 5c, d) (*P*<0.01, *n* = 10 mice each group).

## Discussion

Human and animal rabies cases that are caused by a street rabies virus infection show neuronal dysfunction, such as abnormal neurotransmitter release and ion channel dysfunction, without any evidence of a significant degree of neuronal death. Stein *et al.* (2010) described the viral antigen distribution in the brainstems, cerebella, hippocampi and cerebra in 13 different species, and the hippocampus was suggested as the optimal site for RV detection in dogs and cats. Our study demonstrated that MRV was distributed in the cortices, the thalamuses, the cerebella, and particularly the hippocampi in mice at 7 days p.i.; in agreement with previous reports, no obvious damage to the neuronal soma was detected at this time point. Therefore, the neuronal processes were also examined. Scott *et al.* (2008) found relatively few changes in the perikarya and neuronal processes in the hippocampi of challenge virus (CVS)-infected transgenic mice expressing yellow fluorescent protein (YFP).

Li *et al.* (2005) reported that the apical dendrites in the hippocampi became disorganized in the mice intracerebrally infected with the pathogenic CVS-N2C, a fixed RV strain. In contrast, Scott *et al.* (2008) found relatively few changes in the perikarya and neuronal processes in the hippocampi of CVS-infected YFP mice, and they attributed the discrepancy in these findings to the route of inoculation and the pathogenicity of the strain.

In the present study, no obvious morphological changes were seen in the processes or cell bodies of pyramidal neurons at day 7 p.i., supporting the idea that the fetal rabies virus causes neuronal dysfunction rather than neuronal damage. This is also consistent with the strain-dependent pathogenicity differences that were suggested by Scott *et al.* (2008), since we used a street rabies virus.

Dendritic spines are small membranous protrusions that typically receive inputs from the excitatory synapses of axons. Dendritic spines contain neurotransmitter receptors, organelles and signalling systems that are essential for synaptic function and plasticity, and numerous brain disorders are associated with abnormal dendritic spines (Hutsler & Zhang, 2010; Glantz & Lewis, 2000). Despite the presence of severe neuronal dysfunction in rabies cases, few studies have focused on the dendritic spine alterations caused by rabies. Studies have showed dendritic morphological alterations of cortical pyramidal neurons (including loss of dendritic spines) with RV infection (Torres-Fernández *et al.*, 2007). Our results showed a significant decrease in the number of dendritic spines in certain brain regions of rabid mice *in vivo* and *in vitro*. This decrease may partially explain why RV infection causes neuronal dysfunction rather than neuronal damage.

The cytoskeleton plays an important role in intracellular transport, cellular division and cell shape. The cytoskeleton of a dendritic spine is primarily made of F-actin. Dynamic changes in F-actin have been found in herpes simplex virus 1-infected human neuroblastoma cells (Xiang *et al*., 2012). In the case of RV, alterations in the actin-based cytoskeleton have been described in the neuroblastoma cells by Ceccaldi *et al.* (1997), who found that the nucleocapsid of the virus had no direct action on the kinetics of actin polymerization, but could inhibit the actin-binding effect induced by dephosphorylated synapsin I (Bloom *et al.*, 2003; Ceccaldi *et al.*, 1997; Valtorta *et al.*, 1992). Synapsin I is known to interact with the actin-based cytoskeleton to induce the release of neurotransmitters and the recycling of synaptic vesicles. Thus, we sought to determine if the decrease in the number of dendritic spines was associated with changes in F-actin. We analysed the levels of F-actin in the stratum radiatum of the CA1 region and found that an RV infection causes the depolymerization of F-actin. A number of proteins that regulate the actin cytoskeleton have been identified, including cofilin (also named actin depolymerization factor) (Hotulainen *et al.*, 2005; Kiuchi *et al.*, 2007), profilin, gelsolin, drebrin and CaMKII (Ackermann & Matus, 2003; Bamburg, 1999; Ivanov *et al.*, 2009; Lisman *et al.*, 2002; Nag *et al.*, 2009). Cofilin can depolymerize F-actin through binding at the interface between the actin monomers. Cofilin is inactivated by phosphorylation; therefore, the dephosphorylation of cofilin at the Ser3 residue leads to cofilin activation and the subsequent depolymerization of F-actin. However, our study showed an increase in phosphorylated cofilin, which may be attributed to the compensatory mechanisms associated with F-actin dynamics (Shi & Ethell, 2006).

In our study, we have determined that an RV infection can decrease the number of dendritic spines *in vivo* and *in vitro*. We also showed that this decrease may be partially caused by F-actin depolymerization. Future work will analyse the functional significance of these cytoskeletal and synaptic changes.

## Methods

### 

#### Animals and viruses.

The ICR (imprinting control region) mice (body weight: 8–12 g) used for the experiments were purchased from the Changchun H and N Animal Breeding Center for Medicine and were fed and handled under the guidelines of the Animal Care and Use Committee of Jilin University. The MRV strain (GenBank accession no. DQ875050.1) of street rabies virus, which is a canine rabies virus variant, isolated from the Henan province of China, was grown in mouse N2a neuroblastoma cells. The mice were infected with MRV by intracranial inoculation at a dose of 10 MIC (minimum inhibitory concentration) LD_50_ per 30 µl. Most of the mice in the late stage of infection exhibited furious rabies, manifested by behaviour including agitation and seizures. In line with the criteria reported previously (Laothamatas *et al.*, 2008), we referred to mice exhibiting furious rabies as furious rabid mice.

#### Co-culture of primary hippocampal and cortical neurons.

As previously described, the primary neuronal cultures were derived from the cerebral cortices and the hippocampi of mice on embryonic day 16.5 (E16.5) (Fath *et al.*, 2009). The cortices and hippocampi were incubated with 0.25 % trypsin (Invitrogen), and 50 µl of 10 mg ml^−1^ DNase I stock (Invitrogen) was added for 30 s to break down the DNA and avoid tissue clumping. The cells were then titrated in glass pipettes. The cell suspension was adjusted to the appropriate concentration to obtain 1×10^3^ living cells from the hippocampus and 1×10^5^ living cells from the cortex per 75 µl Dulbecco’s modified Eagle’s medium (DMEM; Invitrogen) supplemented with 10 % FBS (Invitrogen). The dissociated neurons were initially plated in 10 % FBS/DMEM on poly-d-lysine-treated (0.1 mg ml^−1^; Sigma) coverslips with wells. A total of 75 µl of the hippocampal cell suspension was directly plated onto a coverslip, and 75 µl of the cortical cell suspension was slowly plated in a ring surrounding the hippocampal cells. After 2 h, the plating medium was carefully replaced with equilibrated neurobasal media containing B27 supplement (Gibco) and 2 mM l-glutamine (Invitrogen). The cells grew for up to 4 weeks without any further change of medium. The cells were infected with MRV for 120 h at 10 TCID_50_ per well and then fixed with 4 % paraformaldehyde (Invitrogen) and mounted for subsequent fluorescence staining.

#### Immunohistochemistry for RVBV antigen.

As previously described (Li *et al.*, 2007), the MRV-infected mice (*n* = 15) and the control mice (*n* = 10) were anaesthetized with 50 mg kg^−1^ pentobarbital (Invitrogen) and then intracardially perfused with 50 ml PBS followed by 50 ml of freshly prepared 4 % paraformaldehyde in 0.1 M phosphate buffer (pH 7.4; Invitrogen) at day 4 p.i. and day 7 p.i. The brain was dissected and post-fixed in the same fixative for 2–6 h at 4 °C. The brains were then washed in a series of cold sucrose solutions of increasing concentration. The samples were embedded in an OCT (optimal cutting temperature) compound (Tissue-Tek; Sakura Finetek Japan), frozen on dry ice and cut into transverse sections at a thickness of 40 µm using a freezing microtome (Leica). Immunostaining was subsequently performed on these free-floating sections. The cryosections were incubated with 0.3 % H_2_O_2_ and blocked with 3 % normal goat serum (Invitrogen) in 0.1 M phosphate buffer for 30 min at room temperature; the cryosections were then incubated with a rabbit polyclonal anti-RV antibody (1 : 200 dilution; made and stored in our laboratory) overnight at 4 °C. Next, the sections were incubated with HRP-labelled anti-rabbit IgG (Sigma) for 1 h at room temperature and washed and developed with 3,3′-diaminobenzidine (DAB; Sigma). The stained sections were examined using an Olympus IX 51 microscope.

#### Histopathology.

The perfused mice brains were dehydrated through a graded series of ethanol and embedded in paraffin. Paraffin sections of 4 µm thickness were prepared and stained with haematoxylin and eosin (H&E; Sigma).

#### Immunofluorescence.

The animals and cultured cells were fixed or sectioned, as described above. After blocking with a normal 3 % goat serum in 0.1 M phosphate buffer containing 0.1 % Triton X-100 (Sigma) for 30 min at room temperature, the cryosections were incubated with Alexa Fluor 488 phalloidin conjugate (2 mg ml^−1^; Invitrogen) for 45 min at room temperature. Some sections were incubated with a polycolonial anti-rabbit MAP2 antibody (1 : 200; Abcom) and anti-human RABV antibody (1 : 200; stored in our laboratory), the cultured cells were incubated with anti-MAP2 antibody and Alexa Fluor 488 phalloidin conjugate in 0.1 M phosphate buffer overnight at 4 °C. After being washed with 0.1 M phosphate buffer, sections were reacted with Alexa 594-conjugated goat anti-rabbit IgG antibody and Alexa 488-conjugated donkey anti-human IgG antibody. Next, the cryosections and the cells were mounted onto glass slides with Immu-Mount (Thermo) and examined with a laser scanning confocal microscope (Olympus FV1000) under an excitation wavelength of 488 or 594 nm.

#### Western blot analysis.

To analyse the cofilin and actin changes that were caused by the MRV infection, brains were collected from the rabid and the control mice. The hippocampi were then individually dissected and homogenized in an SDS-PAGE sample buffer containing 3 % SDS, 2 % β-mercaptoethanol and 5 % glycerol in 60 mM Tris buffer (pH 6.7), as previously described (Ouyang *et al.*, 2005, 2007). The homogenized samples were boiled for 5 min and stored at −20 °C. The protein concentration was determined by the Lowry method. Thirty micrograms of protein were separated by 15 % SDS-PAGE and transferred to polyvinylidene difluoride membranes. After incubation with a primary antibody (1 : 1000; Cell Signalling) that recognized phosphorylated cofilin (p-cofilin) at Ser3, the membranes were incubated with a peroxidase-conjugated secondary antibody and visualized with an ECL detection kit (Pierce). The blots were reprobed for total cofilin (1 : 1000; Cytoskeleton) and actin (1 : 500; Sigma). The signals were scanned for quantitative analysis with ImageJ.

The ratio of F-actin to G-actin was measured by previously published Western blotting techniques (Gu *et al.*, 2006). Briefly, the hippocampi from the control and rabid mice were isolated and homogenized in a cold lysis buffer (10 mM K_2_HPO_4_, 100 mM NaF, 50 mM KCl, 2 mM MgCl_2_, 1 mM EGTA, 0.2 mM dithiothreitol, 0.5 % Triton X-100, 1 M sucrose, pH 7.0) and then centrifuged at 15 000 ***g*** for 30 min. The supernatants were used for measuring soluble actin (G-actin). For F-actin, the pellets were resuspended in a lysis buffer and an equal volume of 1.5 M guanidine hydrochloride, 1 M sodium acetate, 1 mM CaCl_2_, 1 mM ATP and 20 mM Tris/HCl, pH 7.5. The pellets were then incubated on ice for 1 h with gentle mixing every 15 min to depolymerize the F-actin. The samples were centrifuged at 15 000 ***g*** for 30 min, and the supernatants were used to measure the insoluble F-actin. Samples from the supernatant (G-actin) and pellet (F-actin) fractions were proportionally loaded and analysed by Western blotting.

#### Quantitative analysis.

To analyse the F-actin levels in the control and MRV-infected groups, all of the sections were scanned with an Olympus FV1000 laser scanning confocal microscope under the same parameters. The fluorescence intensity of F-actin in cryosections was calculated by an experimenter who was blinded to the treatment conditions. For the *in vitro* experiment, 10–15 neurons were randomly selected, and the mean number of dendritic spines per 10 µm of dendrite was calculated. A Student’s *t*-test was used to determine the statistically significant differences in the dendritic spine number, the F-actin intensity, and the quantified protein expression between the control and the infected groups. All of the data were expressed as the means±sd. Statistical significance was defined as *P*<0.05.
